# Application of a MEMS-Based TRNG in a Chaotic Stream Cipher

**DOI:** 10.3390/s17030646

**Published:** 2017-03-21

**Authors:** Miguel Garcia-Bosque, Adrián Pérez, Carlos Sánchez-Azqueta, Santiago Celma

**Affiliations:** Group of Electronic Design, University of Zaragoza, 50009 Zaragoza, Spain; aprz@unizar.es (A.P.); csanaz@unizar.es (C.S.-A.); scelma@unizar.es (S.C.)

**Keywords:** cryptography, MEMS accelerometer, true random number generator, skew tent map, stream cipher

## Abstract

In this work, we used a sensor-based True Random Number Generator in order to generate keys for a stream cipher based on a recently published hybrid algorithm mixing Skew Tent Map and a Linear Feedback Shift Register. The stream cipher was implemented and tested in a Field Programmable Gate Array (FPGA) and was able to generate 8-bit width data streams at a clock frequency of 134 MHz, which is fast enough for Gigabit Ethernet applications. An exhaustive cryptanalysis was completed, allowing us to conclude that the system is secure. The stream cipher was compared with other chaotic stream ciphers implemented on similar platforms in terms of area, power consumption, and throughput.

## 1. Introduction

Due to the necessity of encrypting high amounts of data in real time, there has been an increasing interest in cryptography in the last decades. Usually, stream ciphers are used when a high encryption speed is required or when the amount of data is unknown. In a stream cipher, bits or bytes are encrypted individually and it is not necessary to store blocks of data before their processing, as typical block ciphers do because they cannot directly cipher blocks that are shorter than their block size. Because of this fact, stream ciphers usually have less stringent memory requirements than other kinds of ciphers, which results in cheaper implementation in certain applications such as Internet of Things (IoT) devices in wireless sensors networks (WSN). In these ciphers, a seed (key) is used in order to generate a pseudorandom sequence (keystream). Each bit of this sequence is then combined with a bit from the message using an XOR operation. To decrypt the message, an identical system with the same seed is used to generate a duplicate keystream that is XORed with the encrypted message.

Some of the most promising stream ciphers use chaotic maps to generate the keystream. The main advantage of these systems is that, due to their chaotic nature, they have the properties of ergodicity: high sensitivity to the initial conditions and random-like behavior that are related to the properties of confusion and diffusion that are considered to be essential in any cryptosystem [1]. Therefore, they are usually capable of generating sequences with good random properties and a high sensitivity on the key. Their main disadvantage is that these algorithms usually include some multiplications and divisions that increase their complexity compared to other stream ciphers based on modular arithmetic.

On the other hand, it has been proven that, in order to be secure, stream ciphers should not use the same key to encrypt several messages [2]. Furthermore, since the key should be difficult to guess by an attacker, it is not advisable to use a predictable algorithm or a person to generate it. Therefore, it is preferred to generate the key or seed using a True Random Number Generator (TRNG). In this work, a chaos-based stream cipher that uses a MEMS (microelectromechanical system) accelerometer as a seed generator is proposed. Our motivation for using a MEMS accelerometer as a TRNG comes from the fact that previous studies have shown that these devices are capable of generating good random numbers [3]. Although other elements such as silicon resistors can also be used as TRNGs, they typically require a big silicon area in order to be implemented and quite complex post-processing [4,5]. Furthermore, MEMS accelerometers are cheap and are ubiquitously present in many wireless devices (such as smartphones, wearable devices, laptops, drones, etc.), so it is possible to reuse an element that is already available in the device [6]. In [7], a chaos-based cryptosystem with a sensor based seed generator was preliminary advanced. However, in this work, the seed generation block is analyzed in much more detail. Furthermore, a different stream cipher based on a hybrid algorithm mixing Skew Tent Map and Linear Feedback Shift Register proposed in [8] has been adapted for this application. The algorithm proposed in [8] has been improved, obtaining a higher throughput while maintaining the same level of security.

**Figure 1:**
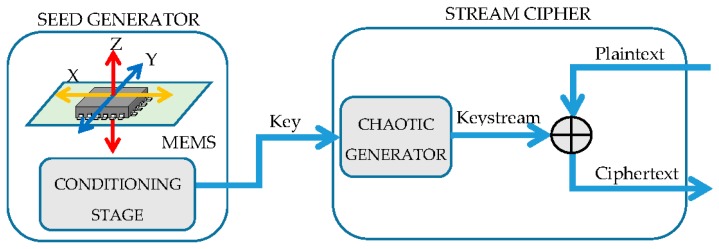
Overview of the full cryptosystem, including a chaos-based stream cipher and a sensor-based seed generator.

A scheme of the proposed cryptosystem is shown in Figure 1. The proposed cryptosystem uses a seed generator block composed by a commercially available accelerometer [9] and a conditioning stage. Both systems are able to generate enough randomness, as is proven in the next sections. Using these seeds, a chaotic stream cipher based on a Skew Tent Map (STM) and a Linear Feedback Shift Register (LFSR) generates a keystream K that is XORed with the plaintext P in order to obtain the ciphertext C. The whole stream cipher block has been implemented and tested in a Field Programmable Gate Array (FPGA), obtaining a throughput of 1.072 Gbps. Regarding the seed generation, an in-depth analysis of the viability of the system has been made. This analysis includes the capture of the accelerometer signal at different sample rates and a study of its randomness properties. Furthermore, a conditioning stage based on Secure Hash Algorithm 512 (SHA-512) [10] has been implemented in order to whitening the raw sensor data.

The paper is organized as follows: Section 2 explains the stream cipher algorithm in detail, as well as its implementation results and a comparison with other chaos-based stream ciphers; Section 3 includes an analysis of the noise signal generated by a commercial accelerometer at rest as well as an explanation of the conditioning stage; Section 4 presents a cryptanalysis of the encryption system; finally, conclusions are drawn in Section 5.

## 2. Stream Cipher Algorithm

### 2.1. Skew Tent Map

**Figure 2:**
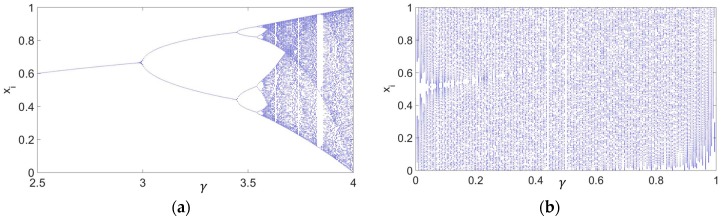
This figure represents several consecutive values of a sequence $x_{i}$ generated by different values of the chaotic parameter γ for: (**a**) The Logistic Map; (**b**) The Skew Tent Map.

Most of the chaotic systems only present chaos for a certain set of initial parameters while other initial parameters lead to a periodic behavior that is not suitable for secure applications. The set of parameters that produces a chaotic behavior is not usually within a continuous region and it is often unknown [11]. The Logistic Map, despite being used in many of the proposed chaotic cryptosystems [12,13], presents this problem. As an example, a bifurcation diagram of the Logistic Map, which represents several consecutive values of the sequences $x_{i}$ as a function of the chaotic parameter γ, is shown in Figure 2a. Most values of γ>3.57 exhibit chaotic behavior but there are some regions in white where the system presents a periodic behavior. Moreover, even in the chaotic regions there exist an infinite number of initial conditions that lead to periodic cycles [14]. Several methods have been capable to find some of the periodic windows [15,16], but the whole set of parameters that produces periodic windows remains unknown. Therefore, it can be difficult to define the key space in this type of systems.

In our work, we based our encryption algorithm on a Skew Tent Map since it is one of the simplest chaotic systems with a continuous region where all the parameters are capable of generating a chaotic (non-periodic) sequence. The Skew Tent Map (Figure 2b) [17] is given by

(1) $$f\left(x_{i}\right)=x_{i+1}=\{\begin{array}{c} x_{i}/\gamma \;x_{i}\in \left[0,\gamma \right]\\ \left(1-x_{i}\right)/\left(1-\gamma \right)\;x_{i}\in \left(\gamma ,\;1\right] \end{array}$$

where $x_{0}$ is the initial value of the $\left\{x_{i}\right\}$ sequence and $\gamma ,\;x_{0}\in \left(0,1\right)$. For any value of $x_{0}$ and γ, the map exhibits a chaotic behavior, which makes it a good choice for a cryptosystem. Moreover, this algorithm is quite simple although the division operation can be difficult to implement in some platforms such as FPGA. Therefore, we suggest that the values of 1/γ and 1/(1−γ) should be calculated and saved and the successive values of $x_{i}$ should be obtained using multiplications instead.

### 2.2. Increasing the Period Length

When the STM is implemented with a precision of n bits, there are only $2^{n}$ possible values for each $x_{i}$ and, for a given γ, each $x_{i}$ determines the next value $x_{i+1}$. Therefore, the sequence will become periodic and its maximum period will be $2^{n}$. However, the typical cycle lengths are found experimentally to be much shorter. In order to increase the period, and therefore the security, of these sequences, it is possible to slightly change the values of each $x_{i}$ $\left(x_{i}\to {\tilde{x}}_{i}\right)$ and use this modified ${\tilde{x}}_{i}$ to obtain the next value of the sequence as $x_{i+1}=f\left({\tilde{x}}_{i}\right)$. In our cryptosystem, the values of ${\tilde{x}}_{i}$ are obtained by XORing the least significant bit (LSB) of each $x_{i}$, $x_{i}^{0}$, with the least significant bit of an LFSR, $y_{i}^{0}$. Using this method, the period of the sequence can be set up to be above a given value using the following proposition, proven in [18].

Proposition 1: Given the binary sequences a(k), b(k) and c(k)=a(k)⊕b(k), for k∈ℕ and $P_{a}$, $P_{b}$ and $P_{c}$ their respective periods. If $P_{b}$ is prime, then $P_{c}\geq mP_{b}$ with m≥1.

Therefore, if we consider c(k) as the LSB of ${\tilde{x}}_{i}$, ${\tilde{x}}_{i}^{0}$, a(k) as the LSB of $x_{i}$, $x_{i}^{0}$, and b(k) as the output bits, $y_{i}^{0}$, of an LFSR with a prime period $P_{b}$, the period of the $\left\{{\tilde{x}}_{i}\right\}$ sequence will be $P_{c}\geq P_{b}$. In our work, a 61-order LFSR has been used, which gives us a period of, at least, $2^{61}$. This choice guarantees that the period is big enough for our purposes (a sequence with a period of $2^{61}$ transmitted at a speed of 1 Gbps would take more than 73 years to repeat itself).

### 2.3. Stream Cipher Algorithm

**Figure 3:**
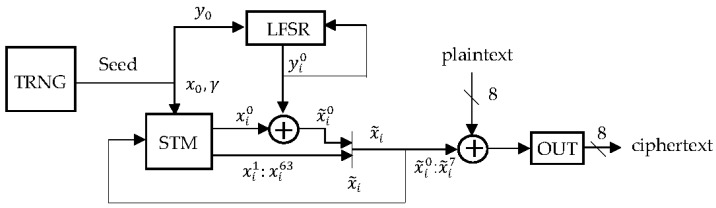
Block diagram of the proposed cipher system.

**Figure 4:**
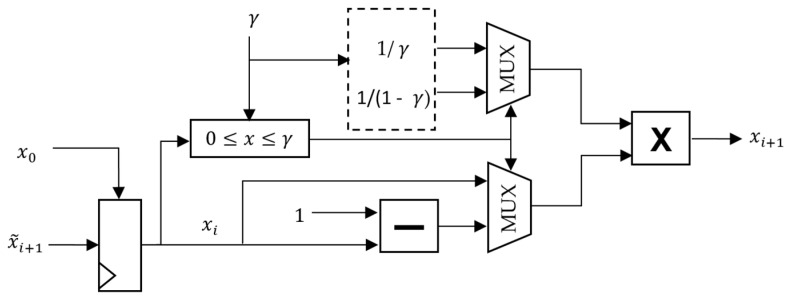
Block diagram of the proposed 64-bit Skew Tent Map generator. Values 1/γ and 1/(1−γ) are precalculated when initializing the chaotic cipher and they are constants for the whole communication session until a new key is requested.

The schematic of the proposed stream cipher is shown in Figure 3. The principle of operation is as follows: first, the accelerometer is used to generate the initial parameters of the STM, $\gamma ,\;x_{0}$ and the initial state of the LFSR, $y_{0}$. Then, the encryption algorithm generates a pseudorandom sequence $\left\{{\tilde{x}}_{i}\right\}$ using the STM algorithm (Figure 4) and XORing the LSB of each $x_{i}$ from the STM, $x_{i}^{0}$, with a pseudorandom bit generated by the LFSR, $y_{i}^{0}$. Furthermore, in order to increase the security of our system, we have only used the eight least significant bits from each ${\tilde{x}}_{i}$ $\left({\tilde{x}}_{i}^{0},\;{\tilde{x}}_{i}^{1},\;\ldots ,{\tilde{x}}_{i}^{7}\right)$ to encrypt the message. This way, we restrict the amount of information that an attacker could obtain, so that some attacks such as a chaotic reconstruction of the map are harder to accomplish. Therefore, the message is divided in 8-bit blocks, $m_{i}$, and, after each iteration, these bits are combined with eight bits from ${\tilde{x}}_{i}$ with an XOR gate.

The receiver is also composed of STM and LFSR blocks. By using the same initial parameters that were used to encrypt the message, the receiver is capable of recovering the original message by combining its sequence with the encrypted sequence with an XOR gate. In our tests we have directly introduced the same initial parameters in the transmitter and the receiver although, in a real implementation, the initial parameters should be generated in the transmitter and sent to the receiver using a secure channel. This could be easily accomplished using secure asymmetric algorithms such as Rivest, Shamir and Adleman (RSA).

## 3. Seed Generator

### 3.1. Noise Signal Analysis

**Figure 5:**
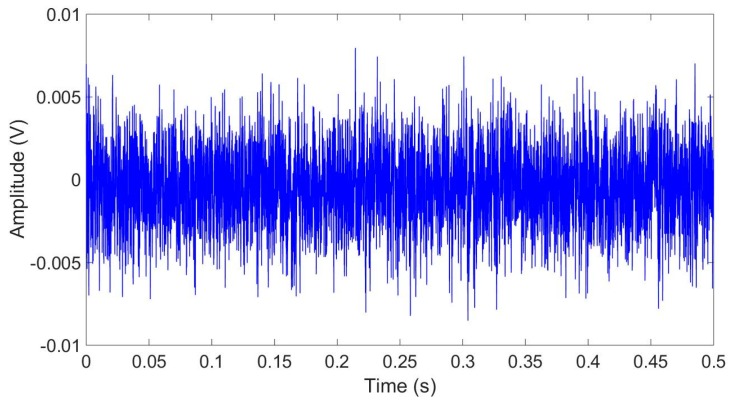
Sample random data generated by the accelerometer at rest, measured using a sample rate of 25 kSps.

In order to generate values for the initial parameters of the STM, γ and $x_{0}$, as well as the initial state of the LFSR, $y_{0}$, the feasibility of a TRNG based on the capture of noise produced by an accelerometer at rest was studied. Simulations have been done to test the proposed method. To generate random numbers, the noise signal produced by means of the evaluation board EVAL-ADXL335Z has been used. This board contains a small low-energy three-axis accelerometer ADXL335 [9]. This accelerometer is composed by a polysilicon surface-micromachined capacitive sensor and a conditioning electronic stage that implements an open-loop measurement architecture. Acceleration deflects the moving mass and unbalances the differential capacitor resulting in a sensor output whose amplitude is proportional to acceleration. The phase-sensitive demodulation technique was then used to determine the magnitude and direction of the acceleration. Some of its typical applications, such as mobile systems or sports and health devices, are strongly related with IoT. The evaluation board was supplied with batteries to avoid coupling 50 Hz or 60 Hz harmonics from the power supply network. AC signal of the measurements of the acceleration in the X axis have been acquired by an oscilloscope at several sampling rates, 250, 25, 2.5, 0.25, and 0.1 kSps using a dynamic range of ±8 mV and 8-bit resolution. An example of noise signal, obtained using a sample rate of 25 kSps is shown in Figure 5. To minimize the correlated common noise, signals generated by the X and Y sensors have been subtracted.

**Figure 6:**
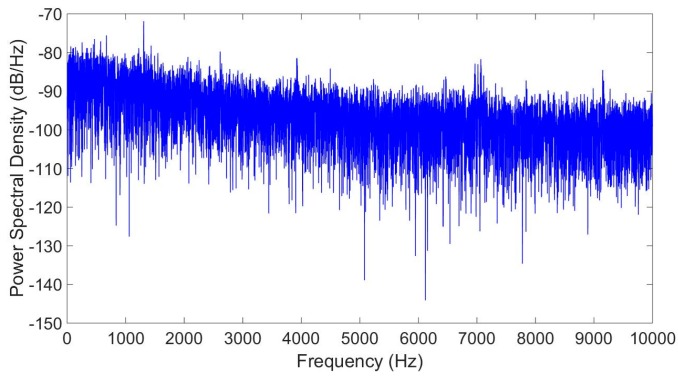
Fast Fourier Transform of the sampled data.

The Fast Fourier Transform (FFT) of this signal has been calculated to analyze the characteristics of the noise (Figure 6). As can be seen, the obtained power spectral density decreases with the frequency for low frequencies but it stays approximately constant (white noise) for high frequencies.

**Figure 7:**
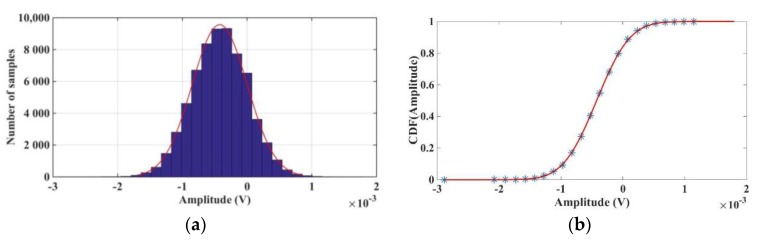
(**a**) Sample data distribution histogram with a normal fit; (**b**) Empirical Cumulative Distribution Function (CDF) (blue) and CDF of a normal distribution (red).

Finally, it was tested if the noise generated by the accelerometer follows a Gaussian distribution, which would be suitable for our application. Figure 7a shows a histogram of the data where it can be seen that the distribution is approximately Gaussian. A normal distribution fit gives a mean of μ=−0.42 mV and a standard deviation of σ=0.43 mV. On the other hand, Figure 7b compares the sensor data cumulative distribution function (CDF) with the standard normal CDF. As can be seen, both cumulative distribution functions are very similar, which indicates that the sensor data is very close to Gaussian noise.

### 3.2. Random Bitstream Post-Processing

**Figure 8:**
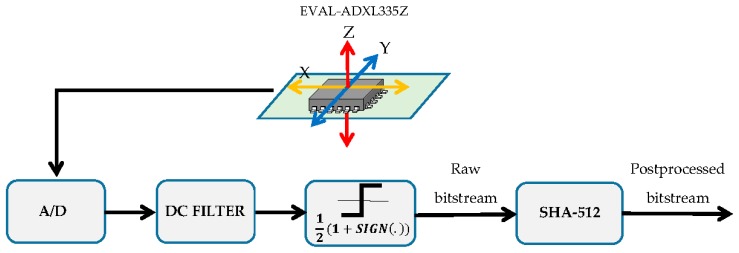
Conceptual block diagram of the processing performed.

After sampling the signal noise, the DC level was eliminated and a sign detection was applied in order to generate a raw bitstream (Figure 8). Once the raw bitstream for key generation was obtained, its viability as a source of random numbers was analyzed. For this purpose, several bitstreams obtained with different sampling rates were subjected to the National Institute of Standards and Technology (NIST) SP 800-22 battery of test [19]. It was observed that none of the bitstream passed all of the NIST tests. However, as the sampling rate decreased, more tests were met, improving the randomness of the bitstream. NIST tests results are shown in Table 1. The list of applied tests with their numeration is the following: Monobit Frequency Test (1), Block Frequency Test (2), Runs Test (3), Longest Runs of Ones (4), Binary Matrix Rank (5), Spectral Test (6), Non Overlapping template Matching (7), Overlapping Template Matching (8), Maurer’s Universal Statistic Test (9), Linear Complexity Test (10), Serial Test (11), Approximate Entropy Test (12), Cumulative Sums Test (13), Random Excursions Test (14), Random Excursions Variant Test (15), Cumulative Sums Test Reverse (16), and Lempel-Ziv Compression Test (17).

According to NIST recommendations, if it is known that the raw noise-source output is biased, appropriate conditioning components can be included in the design to reduce that bias to a tolerable level [20]. Furthermore, several vetted conditioning functions are listed, such as hash-based, cipher-based, and cipher block chaining message authentication code (HMAC, CMAC, and CBC-MAC).

These basic cryptographic primitives are included in many security communication systems, so their use for conditioning randomness sources might not result in extra hardware resources. In case these functions are not available in the communication system, other conditioning techniques can be applied. For example, easier digital techniques such as von Neuman de-biasing [21] can be used. Other zero-bias noise methods such as the one proposed by Bagini-Bucci [22] or Stipčević [23] can be used, although the resulting analogue part of the conditioning stage would be more complex than that presented in this article.

**Figure 9:**
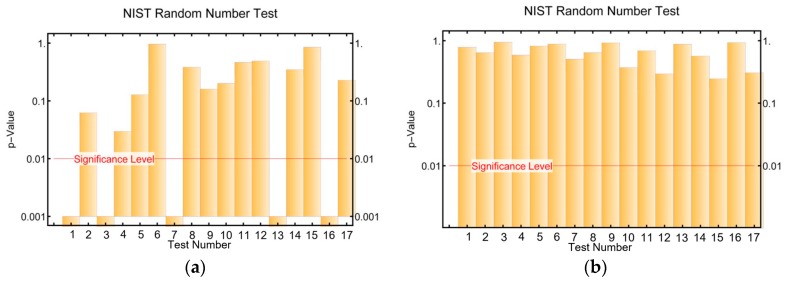
(**a**) NIST test results for bitstream obtained by sampling the sensor at 2.5 kSps; (**b**) NIST test results after applying the SHA-512 algorithm to that bitstream. The test numeration is the same as in Table 1.

In this work, the raw bitstream has been post-processed using the Secure Hash Algorithm 512 (SHA-512) [10] algorithm. The SHA hashing family is one of the most popular techniques among whiteners and is included on NIST listed HMAC conditioners [18]. After post-processing, it has been observed that, at all sampling rates, the NIST randomness tests are passed. As an example, Figure 9 shows the NIST test results before and after post-processing for a sequence obtained at a sample rate of 2.5 kSps.

### 3.3. Mechanical Response of the Accelerometer

Although these measurements were made with the accelerometer at rest, the mechanical response of the accelerometer in an IoT device can often surpass the noise signal. To solve this problem, three methods are proposed.

First, since it is not necessary to generate the keys constantly, the system can generate keys only when the accelerometer is at rest. Currently, some algorithms exist that can detect when the accelerometer is at rest. Although these algorithms are usually used to put the device in sleep mode, they could also be used to activate the key generation process [24].

Second, signal acquisition in inertial MEMS (such as an accelerometer) is usually based on the synchronous demodulation technique, which requires an output low-pass filter to demodulate the output signal and reduce the high frequency noise. Similarly, another path with a high-pass filter could be included. This way, when the device was in a normal operation mode, the low-pass filter path would be used while the system would switch to the patch with the high-pass filter in order to generate the keys.

Third, most of the accelerometers include a Self-Test pin that, when set at a certain voltage, the accelerometer presents a null response. Therefore, this feature could be used to stop the accelerometer from generating the keys. For example, capacitive accelerometers use the force-feedback technique to force the proof mass back to its rest position.

Finally, a combination of these solutions could be used. Further study is needed in order to determine the best solution for each application.

## 4. Implementation Results

The proposed encryption algorithm including the transmitter and the receiver was implemented in a Xilinx Virtex 7 FPGA. In order to test the system, a part of the post-processed bitstream generated by the sensor was used as the initial values of $x_{0},\gamma$ and $y_{0}$. To find a good compromise between encryption speed and security, a 64-bit precision was used. Since γ and $x_{0}$ are within the interval (0,1) they can be implemented easily on the FPGA using a fixed-point arithmetic. The resources used by the transmitter, including the total number of lookup tables (LUTs), slice registers, and digital signal processing blocks (DSPs), as well as the throughput obtained, are shown in Table 2. Furthermore, the area, frequency, and throughput obtained by other chaotic stream ciphers implemented in similar platforms are shown for comparison purposes.

Establishing a comparison between these systems is not straightforward since they have been implemented in different platforms and it is difficult to analyze the level of security that each one provides. For example, the cryptosystem proposed in [27] achieves a higher throughput than the STM-LFSR algorithm but it is transmitting 16 bits instead of eight after each iteration, which may result in a higher vulnerability. However, it can been seen that our system achieves higher performance area ratio (Mbps/slice) than most of the other cipher systems.

**Figure 10:**
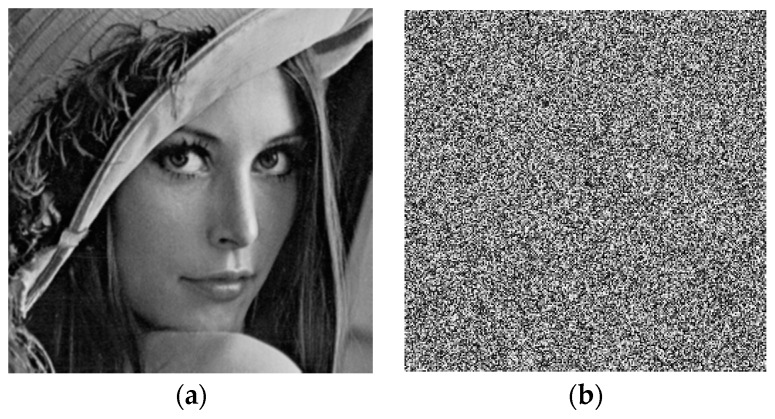
(**a**) Test image; (**b**) Encrypted image.

**Figure 11:**
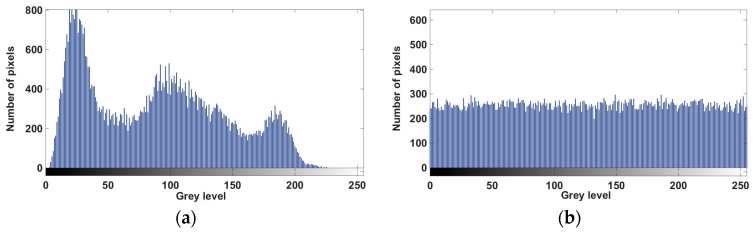
(**a**) Histogram of the test image; (**b**) histogram of the encrypted image.

After implementing the algorithm, we were able to encrypt and decrypt images efficiently. As an example, Figure 10 shows an original and its encrypted images, and Figure 11 shows their respective histograms. The correlation between the original and the encrypted image in this case is only 0.0020, indicating that both images are almost uncorrelated.

Moreover, in the present work, power consumption was also estimated. The FPGA manufacturer power report utilities was used for this purpose. After doing post-implementation simulations, the obtained dynamic power consumption at an operation frequency of 125 MHz (1 Gbps) was 60 mW, which resulted in 60 pJ/bit.

This value is similar to the power consumption of other lightweight ciphers implemented in FPGA, such as [29], where power consumption per ciphered bit is 155 pJ/bit in the AES block cipher and 62 pJ/bit in the Present block cipher. (It should be noticed that these ciphers were implemented in a Xilinx 6 Series FPGA that typically presents a 25%–30% higher power consumption than a Xilinx 7 Series FPGA.) However, these cipher systems achieve throughputs of 116 Mbps and 37.6 Mbps, respectively, which are significantly lower than the throughput achieved by our system.

## 5. Cryptanalysis

### 5.1. Randomness Tests

One of the requirements of a secure cryptosystem is that the output sequence should be undistinguishable from the output of a truly random function for any key used [30]. In order to verify that our stream cipher meets this condition, several sequences generated by different initial conditions were subjected to the NIST tests. Furthermore, in order to see how the inclusion of the LFSR to increase the period length affects the randomness of the sequences, some sequences generated only by the STM were subjected to the same randomness tests. After analyzing several sequences, we concluded that our algorithm was able to generate sequences with good randomness properties and that the presence of the LFSR dramatically increases the randomness properties of the digitized STM.

**Figure 12:**
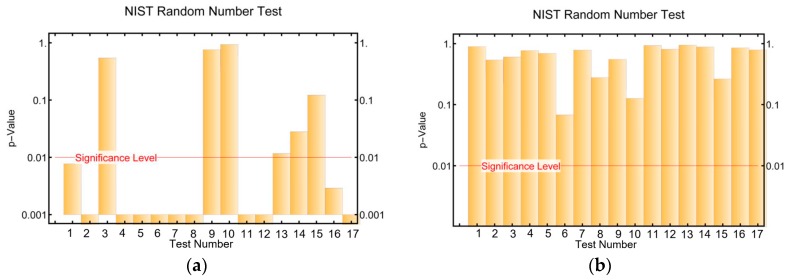
NIST test results for a sequence generated by (**a**) The STM algorithm; (**b**) The STM-LFSR algorithm. The tests numeration is the same as in Table 1.

As an example, Figure 12 shows the NIST results for a particular sequence generated by the STM and the STM-LFSR algorithms respectively. It can be seen that the sequence generated by the STM-LFSR algorithm passes all of them, whereas the sequence generated by the STM only passes seven out of 17 tests.

### 5.2. Parameter Space

In order to analyze the security of our system, the parameter space that, in our system, is given by γ, $x_{0}$, and the initial state of the LFSR, $y_{0}$ should be considered. First, all of the initial parameters should generate sequences with random-like behavior. In our case, the usage of a chaotic system, such as the Skew Tent Map (STM), which is chaotic for any initial parameters chosen in a continuum range $\gamma ,\;x_{0}\in \left(0,1\right)$, and the inclusion of an LFSR, which increases the period length of the sequences, guarantees that our system meets this requirement.

On the other hand, the key space size κ should be big enough to prevent brute-force attacks. Assuming a 64-bit precision and a 61st order LFSR, the total key space size obtained from counting all possible combinations of values of γ, $x_{0}$, and $y_{0}$ is: $\kappa =2^{64+64+61}=2^{189}$. Although some popular lightweight encryption systems such as [31] use 80-bit key sizes, some guidelines recommend that, in order to prevent brute-force attacks in the following years, the key space size should be greater than $\kappa =2^{112}$ [32,33]. Therefore, our key space can be considered to be big enough.

### 5.3. Sensitivity Dependence on the Key

A good cryptosystem should also present a high sensitivity dependence on the parameters to prevent attackers from finding relationships between the parameters and their corresponding masking sequences. Due to the chaotic nature of the STM, similar keys lead to very different sequences, so this condition is satisfied.

In some chaotic systems with several parameters, when sums or subtractions are used, it is possible to fix one of the parameters and find the others using a bit-error-rate (BER) attack [34]. However, this is not possible in the STM since there is a division of the parameters involved.

### 5.4. Sensitivity Dependence on the Plaintext

Our cryptosystem, like most of the stream ciphers, does not present a sensitivity dependence on the plaintext since a single bit change on the plaintext only changes one bit of the ciphertext. This makes the system vulnerable to some attacks, such as a differential known plaintext attack, if several messages are encrypted using the same key. By using a MEMS inertial sensor to generate true random keys, this problem is avoided.

### 5.5. Reconstruction of the Map

A possible attack for some chaotic stream ciphers consists of trying to reconstruct the map by finding the initial parameters via analyzing the output sequence. For example, if an attacker knows the value of $x_{i}$ and $x_{i+1}$, he could use Equation (1) for $f\left(x_{i-1}\right)$ and $f\left(x_{i}\right)$ to calculate the values of γ and $x_{i-1}$.

This method, however, seems to be very difficult to be carry out successfully in our system. First, we are only transmitting eight bits from each $x_{i}$, so the information that a possible attacker has is very limited. Therefore, if an attacker could somehow manage to obtain the last eight bits of $x_{i}$ and $x_{i+1}$, there would still be ${\left(2^{56}\right)}^{2}=2^{112}$ possible values of $x_{i}$ and $x_{i+1}$ that would have to be tried. Furthermore, after each iteration, the LSB of each $x_{i}$ is XORed with a bit generated by an LFSR, which makes the reconstruction even more difficult to accomplish.

Therefore, we can conclude that it is nearly impossible to reconstruct the chaotic map in a reasonable amount of time.

## 6. Conclusions

In this work, a secure stream cipher system was proposed and tested. This system presents a robust STM-LFSR architecture using a MEMS accelerometer with a conditioning stage to generate true random keys. The stream cipher module was implemented in a Xilinx Virtex 7 FPGA using a 64-bit fixed point arithmetic. The stream cipher algorithm was implemented for a throughput of 1.072 Gbps using 805 LUTs, 40 registers and 16 DSPs.

For the seed generation block, the usage of a MEMS accelerometer was studied. We proved that the noise generated by this inertial MEMS accelerometer at rest has random properties, especially when it is sampled at a low sampling rate. However, in order to pass all of the NIST tests, a conditioning stage is necessary. In this work, SHA-512 function was used for this purpose, getting random sequences that pass the NIST test.

An in-depth cryptanalysis was made studying different aspects of our cryptosystem such as randomness, sensitivity dependence on the key and the plaintext, key space, and chaotic reconstruction of the map. This study allows us to conclude that our system can be considered secure, i.e., the amount of computation time needed to hack the cipher is impracticable with current computational equipment and known cryptanalysis algorithms.

On the other hand, although a MEMS accelerometer was proposed to generate the keys, other MEMS and microsensors, such as gyroscopes, light detectors, magnetic sensors, cameras, etc., which are often present in IoT devices, could have been used instead or in a combined way. It is necessary to take into account that in real designs, one must take advantage of the presence of several entropy sources to guarantee the generation of true random numbers. Relating this fact with the low area and high security of our system, we can conclude that this system is suitable for IoT applications.

## Figures and Tables

**Table 1:** NIST test results for bitstreams obtained at different sample rates.

Bitstream Type	Raw Bitstream					Postprocessed Bitstream
Sampling (kSps)	250	25	2.5	0.25	0.1	All
NIST Test						
1	x	x	x	x	x	√
2	x	x	√	x	√	√
3	x	x	x	√	√	√
4	x	x	√	√	√	√
5	√	√	√	√	√	√
6	x	x	√	√	√	√
7	x	x	x	√	x	√
8	x	x	√	√	√	√
9	x	x	√	√	√	√
10	√	x	√	√	√	√
11	x	x	√	√	√	√
12	x	x	√	x	√	√
13	x	x	x	x	x	√
14	√	√	√	√	√	√
15	√	x	√	√	√	√
16	x	x	x	x	x	√
17	x	x	√	√	√	√

**Table 2:** Comparison with Recent Published Chaos-Based Ciphers.

Device	[25]	[26]	[27]	[28]	This Work
Platform	Virtex 5	Virtex 6	Virtex 6	Virtex II	Virtex 7
Main chaotic algorithm	Logistic Map	Henon Map	Logistic Map	Lorenz System	Skew Tent Map
Key generation	NA	NA	NA	NA	TRNG
LUTs	50	1600	643	2718	805
Registers	48	64	160	791	70
Total DSPs	9	16	16	40 ^1^	16
Freq (MHz)	43.2	25.7	93	15.5	134
Throughput (Mbps)	43.2	25.7	1500	124	1072
Slices ^2^	25	408	181	1755	210
Mbps/slice	1.728	0.063	8.287	0.07065	5.105

^1^ Virtex II uses 18 × 18 multipliers instead of DSPs; ^2^ The number of slices has been estimated from the number of Registers and LUTS, assuming unrelated logic.
